# Supplementary material for: An atomic Fabry–Perot interferometer using a pulsed interacting Bose–Einstein condensate
Source: Sci Rep. 2020 Sep 14;10:15052. doi: 10.1038/s41598-020-71973-0 (PMC7490404; doi:10.1038/s41598-020-71973-0)
Supplement: Supplementary file 1 — Supplementary Information [file 41598_2020_71973_MOESM1_ESM.pdf]

An atomic Fabry-Perot interferometer using a pulsed interacting Bose-Einstein condensate: Supplementary Information

P. Manju *et al.*

An atomic Fabry-Perot interferometer using a pulsed interacting Bose-Einstein condensate: Supplementary Information

P. Manju,[1] K. S. Hardman,[1] P. B. Wigley,[1] J. D. Close,[1] N. P. Robins,[1] and S. S. Szigeti[1]

[1] *Atomlaser and Quantum Sensors Group, Department of Quantum Science, Research School of Physics, The Australian National University, Canberra 2601, Australia*

SUPPLEMENTARY NOTE 1: REDUCTION OF 3D GROSS-PITAEVSKII EQUATION TO NON-POLYNOMIAL SCHRÖDINGER EQUATION

Consider the 3D Gross-Pitaevskii equation describing the macroscopic wave function of a BEC [1]:

$$i\hbar\frac{\partial\Psi(\mathbf{r},t)}{\partial t} = \left[-\frac{\hbar^2}{2m}\nabla^2 + V_{\text{ext}}(\mathbf{r},t) + g|\Psi(\mathbf{r},t)|^2 - i\hbar\frac{K_3}{2}|\Psi(\mathbf{r},t)|^4\right]\Psi(\mathbf{r},t), \tag{1}$$

where V_{ext} is an external trapping potential of the form

$$V_{\text{ext}}(\mathbf{r},t) = \frac{1}{2}m\omega_\perp^2(x^2+y^2) + V(z,t) \equiv V_\perp(x,y) + V(z,t). \tag{2}$$

Here m is the atomic mass and $\omega_\perp$ is the frequency of the radial harmonic trap. For a sufficiently tight radial confinement (large $\omega_\perp$), we can approximate the wave function as a Gaussian in the radial direction, $\phi(x,y,\sigma(z,t))$, multiplied by a 1D axial wave function $\psi(z,t)$:

$$\Psi(\mathbf{r},t) = \phi(x,y,\sigma(z,t))\psi(z,t) = \frac{\exp\left[-\frac{x^2+y^2}{2a_\perp^2\sigma(z,t)^2}\right]}{\sqrt{\pi}a_\perp\sigma(z,t)}\psi(z,t), \tag{3}$$

where $a_\perp = \sqrt{\hbar/(m\omega_\perp)}$, $\sigma(z,t)$ encodes the width of the radial Gaussian wave function, and $\psi(z,t)$ is normalised to the atom number, $N(t)$ (atom number can vary with time due to three-body recombination losses). Multiplying Eq. (1) by $\phi^*(x,y,\sigma(z,t))$ and integrating over x and y gives,

$$\begin{aligned} i\hbar\frac{\partial\psi(z,t)}{\partial t} = \Bigg[& -\frac{\hbar^2}{2m}\frac{\partial^2}{\partial z^2} + V(z,t) + g\left(\int dxdy\,|\phi(x,y,\sigma(z,t))|^4\right)|\psi(z,t)|^2 - i\hbar\frac{K_3}{2}\left(\int dxdy\,|\phi(x,y,\sigma(z,t))|^6\right)|\psi(z,t)|^4 \\ & + \left(-\frac{\hbar^2}{2m}\int dxdy\,\phi^*(x,y,\sigma(z,t))\left(\frac{\partial^2}{dx^2}+\frac{\partial^2}{dy^2}\right)\phi(x,y,\sigma(z,t))\right) \\ & + \left(\int dxdy\,V_\perp(x,y)|\phi(x,y,\sigma(z,t))|^2\right)\Bigg]\psi(z,t). \end{aligned} \tag{4}$$

Noting that

$$\int dxdy\,|\phi(x,y,\sigma(z,t))|^4 = \frac{1}{2\pi a_\perp^2\sigma(z,t)^2}, \tag{5}$$

$$\int dxdy\,|\phi(x,y,\sigma(z,t))|^6 = \frac{1}{3\pi^2 a_\perp^4\sigma(z,t)^4}, \tag{6}$$

$$-\frac{\hbar^2}{2m}\int dxdy\,\phi^*(x,y,\sigma(z,t))\left(\frac{\partial^2}{dx^2}+\frac{\partial^2}{dy^2}\right)\phi(x,y,\sigma(z,t)) = \frac{\hbar^2}{2ma_\perp^2\sigma(x,y)^2}, \tag{7}$$

$$\int dxdy\,V_\perp(x,y)|\phi(x,y,\sigma(z,t))|^2 = \frac{1}{2}m\omega_\perp^2 a_\perp^2\sigma(z,t)^2, \tag{8}$$

we obtain the NPSE Eq. (9) of the manuscript. The parameter $\sigma(z,t)$ is constrained by minimising the Gross-Pitaevskii action functional, yielding $\sigma(z,t)^2 = \sqrt{1+2a_s|\psi(z,t)|^2}$ [2].

[1] F. Dalfovo, S. Giorgini, L. P. Pitaevskii, and S. Stringari, Rev. Mod. Phys. **71**, 463 (1999).
[2] L. Salasnich, A. Parola, and L. Reatto, Phys. Rev. A **65**, 043614 (2002).
